# Isolated Cecal Necrosis: A Case Report and Scoping Literature Review

**DOI:** 10.7759/cureus.99750

**Published:** 2025-12-21

**Authors:** Stefanos K Atmatzidis, Maria D Velikoudi, Anestis A Basios, Nikolaos K Voloudakis, Dimitrios A Chatzelas

**Affiliations:** 1 2nd Department of Surgery, “G. Gennimatas” General Hospital of Thessaloniki, Aristotle University School of Medicine, Thessaloniki, GRC

**Keywords:** acute abdomen, bowel ischemia, exploratory laparotomy, ischemic colitis, isolated cecal necrosis

## Abstract

Isolated cecal necrosis (ICN), is a rare form of ischemic colitis in which ischemic injury is confined to the cecum. Because its symptoms frequently mimic acute appendicitis or other common abdominal pathology, diagnosis is often delayed, leading to significant morbidity and mortality. We present the case of a 72-year-old man who presented with acute right-lower-quadrant abdominal pain, following a syncopal episode during air travel. Computed tomography (CT) did not reveal intra-abdominal organ pathology, but raised the suspicion of acute-on-chronic occlusion of the proximal superior mesenteric artery, along with a previously undiagnosed intrarenal abdominal aortic aneurysm. Exploratory laparotomy showed localized cecal gangrene without perforation, and a right hemicolectomy with temporary abdominal closure was performed. A second-look operation confirmed bowel viability, allowing safe ileocolic anastomosis. Despite appropriate management, the patient suffered an acute myocardial infarction, developed multi-organ failure, and died on the fifth postoperative day. This case highlights an acute-on-chronic ischemic mechanism in a patient with extensive atherosclerosis and underscores the importance of early CT imaging and timely surgical intervention in elderly patients with vascular risk factors presenting with right-sided abdominal pain. We also provide a comprehensive review of the literature on ICN, discussing its pathophysiology, clinical presentation, diagnostic challenges, and management strategies.

## Introduction

Isolated cecal ischemia (ICI), also referred to as isolated cecal necrosis (ICN), is a rare, localized form of ischemic colitis, confined to the cecum [1]. It usually affects elderly patients with cardiovascular disease (CVD), chronic renal failure (CRF), or episodes of systemic hypotension [2,3]. Due to the anatomical distribution of its blood supply, the cecum may be susceptible to ischemic injury in case of systemic hypotension or localized mesenteric vascular compromise [4]. Moreover, because its symptoms are non-specific and often mimic acute appendicitis, diverticulitis, or a neoplastic tumor, diagnosis is frequently delayed until surgical exploration [5,6]. Early recognition is crucial, as progression to transmural necrosis and perforation can result in significant morbidity and mortality [4,7].

We report the case of a 72-year-old man who presented to the emergency department with acute abdominal pain after an episode of syncope and was diagnosed with ICN during exploratory laparotomy. We also provide a scoping review of the relevant literature concerning the pathophysiology, clinical presentation, diagnostic evaluation, and management of this rare pathology. By summarizing current evidence and highlighting the diagnostic challenge, this report aims to enhance physician awareness and improve decision-making in patients presenting with right-lower-quadrant (RLQ) abdominal pain suggestive of ischemic disease.

## Case presentation

A 72-year-old man presented to the emergency department of our hospital with an acute onset abdominal pain. The patient was a German tourist on summer vacation to a Greek island. During his return flight, he felt dizziness, nausea, and sweating, which was followed by loss of consciousness (syncope). He recovered after approximately one minute, but continued to feel weakness and fatigue. Moreover, he started to feel pain in his abdomen (RLQ), which was accompanied by one episode of vomiting. The airplane made an emergency landing at our city’s airport, and the patient was transferred to our hospital.

Upon arriving at our emergency department, approximately three hours had already passed since the syncopal episode. During examination, the patient was fully conscious and oriented, and his vital signs were normal; blood pressure 105/62 mmHg, heart rate 95 beats per minute, temperature 36.3°C, oxygen saturation 96%. The pain was continuous, moderate-to-severe, aggravated with movement or coughing. The patient reported two episodes of non-bilious vomiting and no other symptoms. He was a smoker (25 pack-years), and had a medical history of arterial hypertension, type 2 diabetes mellitus, and dyslipidemia, under medical treatment with an angiotensin-converting enzyme inhibitor, metformin, and statin, respectively.

Clinical abdominal evaluation revealed a soft, non-distended abdomen, with mild localized tenderness at the RLQ, but no guarding or rigidity. During auscultation, bowel sounds were normal. There was no history of constipation or diarrhea, nor did he complain about urinary symptoms. Digital rectal examination was unremarkable. His laboratory results are presented in Table 1. The electrocardiogram demonstrated sinus rhythm, with no evidence of atrial fibrillation or myocardial ischemia.

**Table 1:** Blood test results at the time of presentation at the emergency department WBC: white blood count, NE: neutrophils, RBC: red blood count, PLT: platelets, PT: prothrombin time, INR: international normalized ratio, aPTT: activated partial thromboplastin time, SGOT: serum glutamic-oxaloacetic transaminase, SGPT: serum glutamic-pyruvic transaminase, LDH: lactate dehydrogenase, CPK: creatine phosphokinase, TnT: troponin T, CRP: C-reactive protein, pH: potential of hydrogen, pO2: partial pressure of oxygen, pCO2: partial pressure of carbon dioxide, HCO3: bicarbonate

Blood test	Result	Reference range
WBC (*10^3^ / μL)	17.4	4.3-10.3
NE (%)	83.7	41-73
RBC (*10^6^ / μL)	4.95	4.38-5.77
Hematocrit (%)	44.9	39.5-51
Hemoglobin (gr/dl)	15.1	13.6-17.2
PLT (*10^3^ / μL)	247	140-440
PT (sec)	11.0	10-15
INR	1.0	-
aPTT (sec)	28.7	26-36
SGOT (IU/L)	94	<40
SGPT (IU/L)	47	<41
Urea (mg/dL)	43	0-50
Creatinine (mg/dL)	1.19	0.7-1.2
LDH (IU/L)	257	135-225
CPK (IU/L)	162	38-190
TnT (pg/mL)	14	<14
CRP (mg/dL)	4.5	<0.5
Arterial blood gas analysis		
pH	7.36	7.35-7.45
pO_2_ (mmHg)	87.9	75-100
pCO_2_ (mmHg)	34.1	35-45
HCO_3_ (mmol/L)	16.9	22-26
Base deficit (mmol/L)	-8.1	-2-2
Lactate (mmol/L)	6.5	0.5-1.5

**Figure 1:**
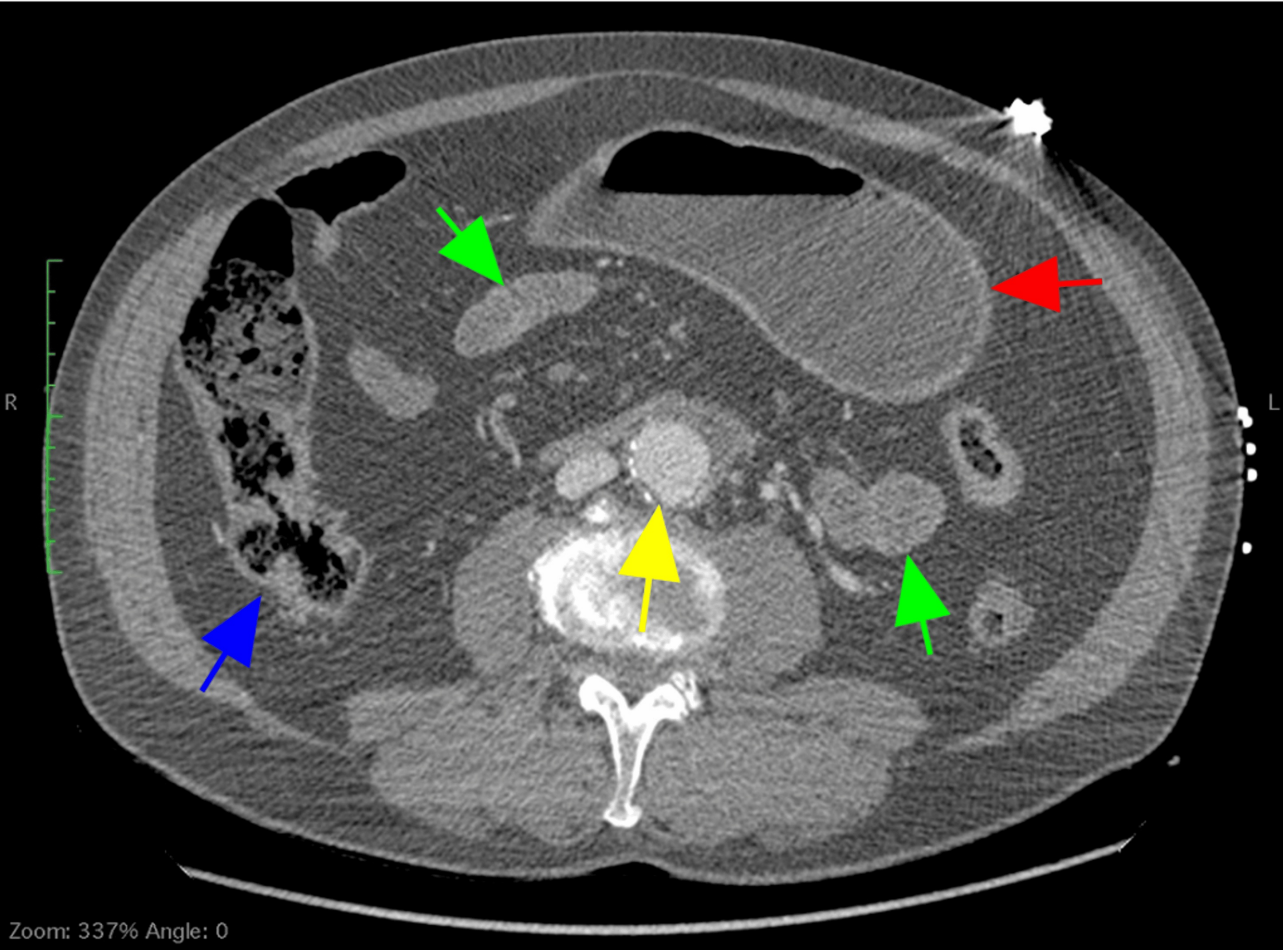
Contrast-enhanced computed tomography scan showing only a mildly dilated stomach (red arrow), and normal appearance of the small (green arrow) and large bowel (blue arrow). An infrarenal abdominal aortic aneurysm is also noted (yellow arrow).

**Figure 2:**
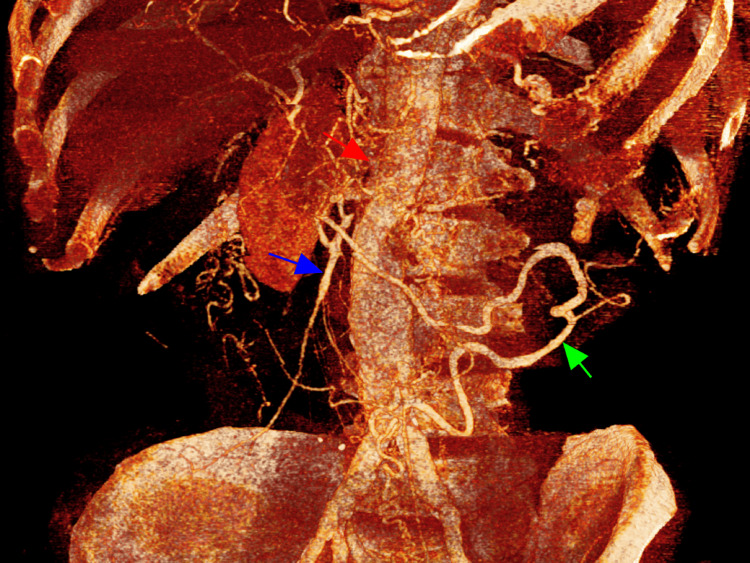
Contrast-enhanced computed tomography scan (3D reconstruction) showing extensive atherosclerosis with total occlusion of the proximal SMA (red arrow), and distal SMA perfusion (blue arrow) through a robust collateral vascular network arising from the inferior mesenteric artery (Arc of Riolan, green arrow). An infrarenal abdominal aortic aneurysm is also noted. SMA: superior mesenteric artery; 3D: three-dimensional

Based on his history, clinical evaluation, and lab results, the Alvarado score was calculated at 8 out of 10; therefore, acute appendicitis was suspected. However, due to the patient’s advanced age and risk factors, we performed a computed tomography (CT) of the abdomen and pelvis after intravenous iodine contrast administration. The CT scan revealed only a mildly dilated stomach, without evidence of wall thickening, pneumatosis, free intraperitoneal air, or obstructive pathology. The small and large bowel, and the appendix, appeared normal in caliber, and no signs of bowel ischemia, intra-abdominal fluid collection, or inflammatory changes were present (Figure 1). However, the CT scan demonstrated significant calcific atherosclerosis and total occlusion of the proximal superior mesenteric artery (SMA). The inferior mesenteric artery (IMA) and celiac axis (CA) remained patent, and there was a vast collateral vascular network between the three vessels, resulting in distal SMA perfusion through an enlarged arc of Riolan (Figure 2). Finally, the CT revealed a previously unknown infrarenal abdominal aortic aneurysm (AAA), of maximum transverse diameter 48mm, without any evidence of rupture or dissection.

The patient was admitted to our department and was commenced on intravenous fluids and broad-spectrum antibiotics. The vascular surgery team was consulted, and suggested the possibility of acute-on-chronic mesenteric ischemia (AMI). In view of this likelihood and the need for direct assessment of the mesenteric circulation and definitive management, an exploratory laparotomy was decided as the most appropriate intervention. 

**Figure 3:**
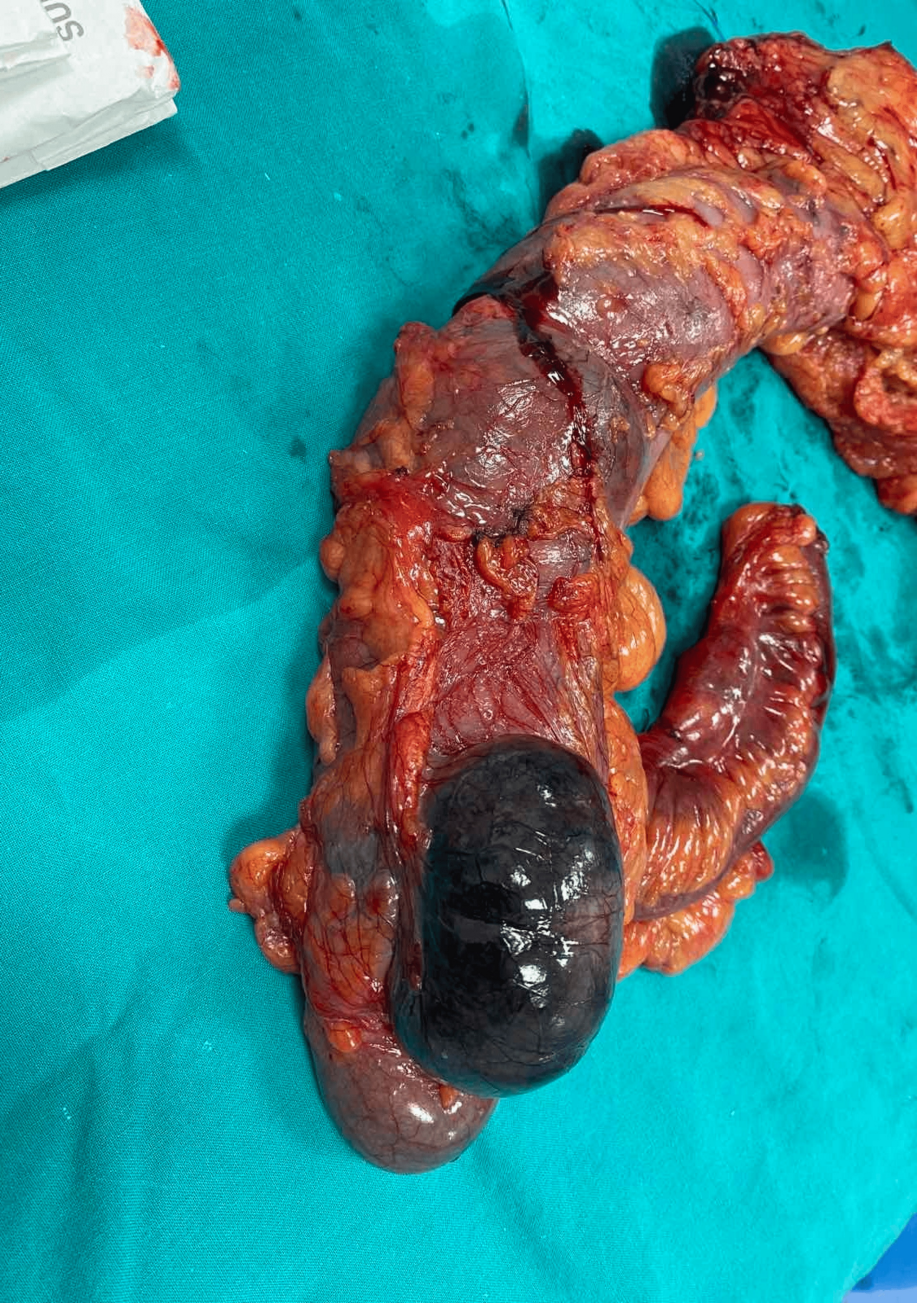
Resected right hemicolectomy specimen demonstrating a demarcated area of gangrene involving the lateral wall of the cecum, while the remaining bowel appears viable

After gaining informed consent, the patient was transferred to the operating room. Just before the operation, 5,000IU of unfractionated heparin was administered intravenously. Up to this time point, approximately three hours had passed since his presentation at the emergency department. Under general anesthesia, a standard midline laparotomy was performed. Intraoperatively, the lateral wall of the cecum was found to be gangrenous, without evidence of bowel perforation (Figure 3). There was some fluid in the right iliac fossa, but the ileum and the rest of the colon appeared healthy. A right hemicolectomy was performed in the standard fashion, without primary bowel anastomosis. Instead, an end ileostomy was constructed, and the abdomen was left open, with temporary placement of a plastic silo (Bogotá bag).

After surgery, the patient was transferred to the intensive care unit (ICU). Twenty-four hours later, he underwent a planned second-look laparotomy to assess bowel viability and exclude progression of ischemia. Intraoperative abdominal inspection revealed healthy-appearing small bowel and remaining colon, with normal color and peristalsis. No additional areas of ischemia, necrosis, or edema were identified. Therefore, a side-to-side stapled ileocolic anastomosis was constructed, and the abdomen was closed in layers, without complications. 

The patient was then returned to the ICU, where he suffered an acute myocardial infarction. Despite optimal medical treatment, his clinical condition gradually deteriorated, with progressive cardiac and respiratory failure, unresponsive to medical therapy. The patient succumbed to multi-organ dysfunction on the fifth postoperative day. The histopathological examination of the resected specimen revealed a partially necrotic cecum, with mucosal denudation, vascular congestion, and areas of mucosal necrosis and ulceration. No features of malignancy or chronic inflammatory disease were noted. 

## Discussion

ICI/ICN is a rare clinical entity within the spectrum of ischemic colitis, characterized by ischemic injury that is confined predominantly or exclusively to the cecum [1]. Although overall colonic ischemia is the one of the commonest forms of intestinal ischemia in adults, focal cecal involvement is rather uncommon [2,3]. ICN is mainly reported in older adults, typically in the sixth to eighth decades of life, with a male predominance [2,3]. Most literature comprises of case reports and small case series, so precise incidence is unknown [1-19].

The cecum, similar to the splenic flexure and rectosigmoid junction, has been described as a “watershed area” in terms of its vascular supply [10,19]. Unlike the splenic flexure and rectosigmoid junction, however, the cecum does not represent an anastomotic zone between two major mesenteric arteries [10,19]. Instead, it receives its blood flow primarily from the anterior and posterior cecal arteries, both considered terminal vessels, which originate from either the ileal or colic branches of the ileocolic artery [10]. In some individuals, the cecum may also receive additional supply from a marginal branch of the right colic artery, or from a recurrent ileal branch, though this occurs in less than 5% of cases [20]. Anatomical variations are common: the posterior cecal artery is absent in up to 10% of individuals, while the recurrent ileal artery is missing in approximately 2% [20]. Rist et al. proposed the “watershed theory," suggesting that when the vascular anastomotic arcade between the anterior and posterior cecal branches is absent, the cecum becomes particularly vulnerable to ischemia, especially at its lateral side [19]. Moreover, the cecum’s relatively large luminal diameter, resulting in lower mural perfusion pressure, and its long vasa recta further contribute to its susceptibility to ischemic injury [4,10].

Two pathophysiological patterns describe ICN pathogenesis [2,4,16]. Firstly, non-occlusive cecal ischemia, which is the most common (25-60%), results from systemic hypoperfusion or regional vasoconstriction [3,5]. In low-flow states, the cecal wall may progress from mucosal ischemia to full-thickness necrosis [3,5,19]. Published series have consistently identified CVD, congestive heart failure, CRF (particularly patients on hemodialysis), diabetes, episodes of systemic hypotension (especially during dialysis or cardiac surgery), and exposure to potent vasoconstrictors (e.g., ergot alkaloids, cocaine, or systemic vasopressors) as common predisposing factors [2-5,13,16,17,19,21]. Secondly, occlusive cecal ischemia, although less common, can arise from true occlusive events, such as embolism or thrombosis of ileocolic branches, that may cause focal cecal infarction [2,4,9]. In practice, many cases reflect a combination of the two mechanisms, such as an atherosclerotic arterial bed that cannot tolerate transient systemic hypotension [9,10,12]. Histopathology commonly shows mucosal and submucosal hemorrhagic infarction and ulceration progressing to transmural necrosis in advanced disease [7,9,11].

In our case, the chronic SMA occlusive disease implies a long-standing adaptation of the mesenteric circulation, with enhanced collateralization from the CA and IMA. Although collaterals can sustain baseline perfusion, they are vulnerable to acute reduction in inflow pressure or to local micro-embolic phenomena [12]. The syncopal episode with transient hypotension that the patient experienced during the flight plausibly precipitated critical hypoperfusion of an already precarious cecal circulation, resulting in focal transmural infarction. Thus, this case likely represents an acute-on-chronic ischemic insult (low-flow decompensation superimposed on chronic occlusive disease), rather than an isolated embolic event limited to ileocolic branches. Indeed, patients with chronic mesenteric disease may develop focal infarction of colon segments when systemic perfusion falls or when competing vascular territories fail [9].

ICN typically presents with acute onset RLQ abdominal pain, which may be accompanied by nausea, vomiting, or low-grade fever; diarrhea or minor rectal bleeding is less common [1,3,4,8,12,15]. Physical examination may mimic acute appendicitis, with localized tenderness and guarding; generalized peritonitis indicates transmural necrosis or perforation [5,6,9,18]. Routine laboratory studies are nonspecific [1,3,4,8,10]. Leukocytosis is common [1,3,4,8,10]. Elevated serum lactate levels can reflect impaired tissue perfusion and anaerobic metabolism, and in the context of acute abdominal pain, raise concern for evolving mesenteric ischemia, even when other laboratory markers remain within normal limits [1,3,4,8,10]. However, neither test reliably excludes nor confirms the diagnosis of AMI [1,3,4,8,10]. Because the clinical presentation overlaps with more frequent surgical emergencies, awareness of risk factors, patient comorbidity, recent hemodynamic status, and laboratory findings is crucial to prompt appropriate imaging and intervention [5,6,9,18]. Therefore, recognition of ICN can present a diagnostic challenge in the emergency department; however, it is essential because late diagnosis with transmural necrosis or perforation substantially increases morbidity and mortality [4,12,13].

Contrast-enhanced CT is the most informative initial imaging modality in suspected ICN [2,8-10]. Characteristic CT findings include focal cecal wall thickening, submucosal edema (target or double-halo sign), pericolic fat stranding, pneumatosis intestinalis (in advanced ischemia), and free intraperitoneal air (if perforation has occurred) [2,8-10]. CT is also useful because it excludes other causes of right-sided abdominal pain, such as acute appendicitis, perforated viscus, or obstructing neoplasm, and can reveal AMI, if present [5,6,9,11,18]. Moreover, CT angiography with its three phases (arterial, portal venous, and delayed) can expeditiously assess the mesenteric circulation, highlighting acute or chronic occlusive phenomena of the mesenteric vessels (both arteries and veins) [22]. Therefore, early CT scanning substantially aids in surgical planning and decision making [9,10,22]. Colonoscopy may be diagnostic when the presentation is subacute, or when the CT suggests a cecal mass rather than frank perforation; mucosal ischemic changes and biopsy allow differentiation from malignancy in mass-forming presentations [11,17]. However, colonoscopy has been reported to increase transmural pressure, leading to decreased colonic perfusion, and is contraindicated in patients with peritonitis or suspected perforation due to the risk of further injury [11,17]. Diagnostic laparoscopy is useful when non-invasive tests are inconclusive [22]. It permits direct inspection, peritoneal lavage, and, when appropriate, therapeutic resection, with reduced morbidity compared with delayed laparotomy in selected centers [4,13,15,23]. A high index of suspicion and combined use of CT, endoscopy (when safe), diagnostic laparoscopy, and exploratory laparotomy are often required for accurate diagnosis [2,4,13,15,22,23].

In our case, the patient presented with acute RLQ pain following a transient syncopal episode. Despite the high Alvarado score suggesting acute appendicitis, our surgical team considered the patient’s age, cardiovascular comorbidities, elevated lactate levels, and syncopal episode indicative of a possible ischemic process. The decision to obtain an urgent contrast-enhanced CT scan proved crucial by revealing chronic proximal SMA occlusion, with collateral-dependent mesenteric perfusion, thus shifting suspicion towards an ischemic etiology, rather than appendicitis. These vascular findings justified the vascular surgeons’ suggestion for early operative exploration, before perforation or generalized peritonitis occurred. Even though no cecal abnormalities were identified on CT, this CT-surgery discrepancy is described in early intestinal ischemia [2,8-10]. Radiologic changes often lag behind the physiological insult, and ischemic injury may progress rapidly from mucosal compromise to transmural necrosis within a short interval, particularly in patients with chronic mesenteric occlusive disease, who rely on fragile collateral networks [2,8-10]. In such cases, CT performed during the early ischemic window may appear normal, before the aforementioned hallmark findings become radiographically apparent [2,8-10]. This case emphasizes the importance of integrating clinical context with risk stratification when evaluating acute abdominal pain in elderly patients, and the diagnostic value of early CT evaluation in elderly patients with right-sided abdominal pain. 

Initial care of patients with ICN focuses on resuscitation: intravenous fluids, hemodynamic optimization, and broad-spectrum antibiotics [4,5,8]. Surgical resection is indicated for transmural necrosis, perforation, or when the diagnosis is established during the operation [4,5,8,10,12]. Surgical procedures range from limited cecal resection/cecectomy to ileocecal resection or standard right hemicolectomy, depending on the extent of ischemia [1-19]. Intraoperative decision between primary anastomosis and diversion (end ileostomy) rests on patient physiology, bowel viability, degree of contamination, and surgeon’s judgment [4,5,8,10,12]. Observational data suggest that diversion is safer in hemodynamically unstable patients, those on dialysis, or with gross abdominal contamination; primary anastomosis may be appropriate in stable patients with minimal contamination [4,5,8,10,12,22]. Conservative (non-operative) management has been reported in carefully selected patients with superficial or subacute ischemic lesions without peritonitis [14,17]. Such patients require close inpatient observation, serial examinations, and endoscopic follow-up, because progression to transmural necrosis can occur [14,17]. However, prospective data on conservative therapy are lacking, and most authors recommend a low threshold for surgical exploration in equivocal cases [22].

In our case, the intra-operative findings demonstrated localized gangrene of the lateral cecal wall with limited peritoneal fluid, but no evidence of perforation or ischemia in the remaining bowel. This sharply demarcated distribution is typical of ICN and aligns with prior reports describing a well-defined, segmental area of cecal infarction (usually the lateral wall), with preservation of the terminal ileum and ascending colon [1,4,8,10,18]. Given the patient’s comorbid status and evidence of localized contamination, a right hemicolectomy with end ileostomy and temporary abdominal closure was performed as a damage-control measure. This staged approach, followed by a planned second-look laparotomy 24 hours later, allowed reassessment of bowel viability before definitive reconstruction. Subsequent exploration revealed a healthy bowel, and a stapled ileocolic anastomosis was safely constructed. Such a two-stage strategy is supported by the literature as an appropriate option in physiologically compromised or high-risk patients, reducing the risk of anastomotic leak, and permitting optimization of hemodynamics, prior to restoration of intestinal continuity [22,24].

Reported morbidity and mortality for ICN vary, reflecting differences in patient comorbidity and delay to intervention [4,5,8,10,12]. Historical series in older, comorbid patients reported substantial perioperative mortality; more recent reports show improved outcomes with earlier imaging and wider use of diagnostic laparoscopy [4,13,15,23]. However, significant morbidity persists in patients with CRF, CVD, or delayed diagnosis, rising to 71-100% [25]. Adverse prognostic factors include advanced age, smoking, multiple comorbid conditions (particularly dialysis and cardiac failure), transmural necrosis with perforation, systemic sepsis, and need for vasoconstrictive support [8-10,12,22,24,25]. A higher leukocyte count or elevated lactate levels at presentation have been associated with transmural disease or perforation, but no single laboratory marker has been found to consistently predict outcome [1,3,4,8,10]. Multi-institutional registries and collaborative series could clarify risk stratification, indications for conservative care, and outcomes. Development of validated clinical prediction tools (incorporating comorbidities, hemodynamic data, laboratory markers, and imaging features) would assist surgical decision-making and might reduce morbidity and mortality. Finally, greater awareness among physicians could enable preventive measures.

In our case, despite a timely diagnosis and technically successful staged surgery, the patient suffered an acute myocardial infarction in the ICU, developed progressive multi-organ failure, and died on the fifth postoperative day. This unfortunate outcome underscores the dominant influence of systemic comorbidity and generalized ischemic burden on prognosis in ICN, rather than the extent of bowel involvement alone. Moreover, postoperative cardiopulmonary deterioration, frequently observed in elderly vascular patients, often reflects the systemic inflammatory response and reperfusion injury following an ischemic insult [22]. This case, therefore, highlights that timely intervention can prevent catastrophic abdominal sepsis, but survival ultimately depends on systemic factors that extend beyond local surgical control.

## Conclusions

ICN is an extremely rare but clinically important, grave cause of acute right-sided abdominal pain. Early recognition, guided by clinical examination, prompt CT imaging, and, when indicated, diagnostic laparoscopy is essential to avoid progression to transmural necrosis and perforation. Surgical resection remains the mainstay for definite treatment. Our case is one of the few published instances of ICN associated with occlusion of the SMA, highlighting an acute-on-chronic ischemic mechanism, precipitated by transient systemic hypotension, in a patient with significant atherosclerotic disease. This combination underscores the vulnerability of the cecum in patients with pre-existing mesenteric vascular compromise and illustrates how even a brief perfusion drop can precipitate localized transmural necrosis, despite an otherwise adequate collateral circulation.
